# Expression of functional alternative telomerase RNA component gene in mouse brain and in motor neurons cells protects from oxidative stress

**DOI:** 10.18632/oncotarget.13049

**Published:** 2016-11-03

**Authors:** Erez Eitan, Admoni Tamar, Grin Yossi, Refael Peleg, Alex Braiman, Esther Priel

**Affiliations:** ^1^ The Shraga Segal Department Microbiology, Immunology & Genetics, Faculty of Health Sciences, Ben-Gurion University of the Negev, Beer-Sheva, Israel

**Keywords:** telomerase RNA component, alternative TERC, telomerase, mouse brain, oxidative stress, Gerotarget

## Abstract

Telomerase, a ribonucleoprotein, is highly expressed and active in many tumor cells and types, therefore it is considered to be a target for anti-cancer agents. On the other hand, recent studies demonstrated that activation of telomerase is a potential therapeutic target for age related diseases. Telomerase mainly consists of a catalytic protein subunit with a reverse transcription activity (TERT) and an RNA component (TERC), a long non-coding RNA, which serves as a template for the re-elongation of telomeres by TERT. We previously showed that TERT is highly expressed in distinct neuronal cells of the mouse brain and its expression declined with age. To understand the role of telomerase in non-mitotic, fully differentiated cells such neurons we here examined the expression of the other component, TERC, in mouse brain. Surprisingly, by first using bioinformatics analysis, we identified an alternative TERC gene (alTERC) in the mouse genome. Using further experimental approaches we described the presence of a functional alTERC in the mouse brain and spleen, in cultures of motor neurons- like cells and neuroblastoma tumor cells. The alTERC is similar (87%) to mouse TERC (mTERC) with a deletion of 18 bp in the TERC conserved region 4 (CR4). This alTERC gene is expressed and its product interacts with the endogenous mTERT protein and with an exogenous human TERT protein (hTERT) to form an active enzyme. Overexpression of the alTERC and the mTERC genes, in mouse motor neurons like cells, increased the activity of TERT without affecting its protein level. Under oxidative stress conditions, alTERC significantly increased the survival of motor neurons cells without altering the level of TERT protein or its activity.

The results suggest that the expression of the alTERC gene in the mouse brain provides an additional way for regulating telomerase activity under normal and stress conditions and confers protection to neuronal cells from oxidative stress.

## INTRODUCTION

Telomerase, a ribonucleoprotein, mainly consists of two catalytic essential elements: a Telomerase reverse transcriptase (TERT) and a Telomerase RNA Component (TERC). TERT re-elongates telomeres by the incorporation of repeated sequences of six nucleotides at the 3′ end of the chromosome using TERC as its template. This activity is essential for the stability of the genome and for the cell lifespan. Telomerase holoenzyme contains additional proteins which are important for its activity, assembly and localization [1–4]. Telomerase is active in proliferative normal cells, embryonic stem cells, in 90% of cancerous cells, and other highly proliferative cells [5], but it is also expressed and active in the brain [6, 7]. The TERC gene varies in length and sequence amongst different species: 148 to 209bp in ciliate, ~1,300bp in yeasts, 390-450bp in vertebrate and in all species these genes are transcribed into a long non-coding RNA. It was demonstrated that the TERC sequence of 35 vertebrates contains eight conserved regions (CR1-CR8), which construct a three domain structure [8]. In addition, the high similarity in the secondary structures of TERC between species, indicates that a conserved structure is essential for telomerase function. In mice the TERC gene is located on chromosome 3 and it is 397 bases long, and as in other vertebrates, the mouse TERC (mTERC) secondary structure contains the core domain consisting of pseudoknot domain and the template, the CR4-CR5 trans-activation domain and the Box H/ACA domain [8]. The template interacts with the 3′ G-strand overhang of telomeres and consists of an 8 nucleotide sequence: CUAACCCU which six of them are reverse -transcribed to generate the telomeric repeats. The pseudoknot is an essential element of TERC and recent studies showed two conformation of the pseudoknot: a triple helix that is critical for telomere elongation and an open conformation which facilitates inactive enzyme (reviewed in [9, 10]). The CR4/CR5 trans-activation domain, like the core domain, interacts directly with TERT. A p6.1 stem loop, which is a highly conserved element among vertebrates, is located in the CR5 region and is essential for the CR4-CR5 assembly, TERT binding and telomerase activity [11, 12]. Furthermore, deletion of the CR5 abolishes telomerase activity *in vivo*. Interestingly, although the p6.1 sequence is completely conserved, studies have shown that the base-pairing rather than the sequence is required for TERT activity [13]. The core domain and the CR4-CR5 domain are in fact the only required TERC elements for *in-vitro* reconstitution of catalytically active telomerase [10]. In addition to its function as the template for TERT, different parts of the TERC molecule together with TERT shape the telomerase catalytic centre, participate in the nucleotide incorporation catalytic activity, are important for the TERT/RNA/Proteins complex assembly, required for the efficient translocation process [14], and also play a key role in transport and regulation of telomerase activity (review in [15]). The importance of TERC expression *in vivo* was demonstrated in several studies: TERC knockout cells and mice exhibited a reduced telomere length with each generation, until reaching a critical shortened length [16, 17]. TERC knockout mice are considered a model of early aging and mice become infertile after 5 or 6 generations [17]. TERC mutations in humans are responsible for the premature aging syndrome Dyskeratosis Congenital (DC) [18]. Recently, both TERT and TERC have been shown to have cellular functions unrelated to telomeres. TERC increased single strand DNA repair by interacting with the DNA kinase KU60 and the Ataxia Telangiectasia Related (ATR) protein [19, 20]. It was also shown that TERC can function as noncoding RNA that protects from apoptosis in CD4 T-cells independently of its function in telomerase activity and telomere maintenance [21].Interestingly, in Arabidopsis two divergent TERC moieties (TER1, TER2) were identified that served as templates for telomerase activity *in vitro* but only TER1 served as telomerase template *in vivo* [22]. However, TERC paralogs have not been reported in other species. Here, we demonstrate the presence of an additional TERC gene (alTERC) in mice which is also located in chromosome 3 and contains a deletion of 18 bp in the CR4 region. Both genes (the TERC and alTERC) are transcribed into RNA *in vivo* (mouse brain and spleen) and *in vitro* in mouse motor neurons like cells. The alTERC interacts with mouse and human TERT and overexpression of either TERC or alTERC enhanced telomerase activity. Under oxidative stress conditions, overexpressing of TERC and alTERC increased the survival of motor neurons like cells without increasing TERT expression or telomerase activity.

## RESULTS

### The mouse genome contains an additional TERC gene

**Figure 1:**
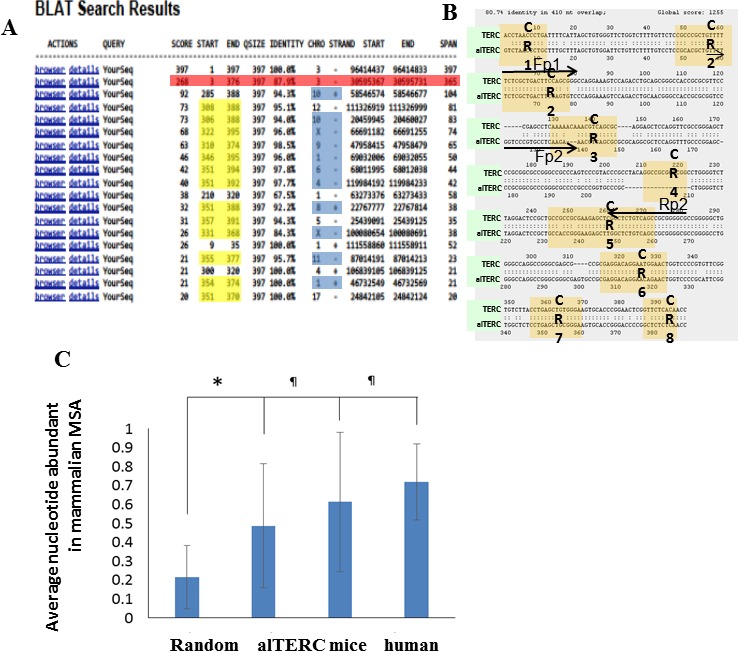
Identification of additional TERC gene in mouse genome using BLAT analysis **A.** BLAT search was performed using the UCSC Genome Browser and the mTERC sequence as a query. Identification of 18 hits, 17 short sequences (20-104 nt) and one long sequence of 365nt (designated alTERC). **B.** Global pairwise alignment to the known mTERC (397nt) revealed 80.7% similarity, deletion of 18nt in the CR4. Arrows represent the location of the designed primers for the amplification by PCR: Fp1 and Rp2 (set 1) for mTERC and Fp2 and Rp2 (set 2) for alTERC. **C.** Comparison of the MSA consensus sequence of TERC to alTERC and to randomly generated sequences of the same length and nucleotide composition.

Searching the Mouse genome by BLAT (BLAST like alignment tool) using the UCSC Genome Browser and the mTERC sequence as a query, revealed a list of 18 hits: 17 of these hits represent short sequences of 20-104 bp (Figure 1A). Interestingly, 11 are followed by telomere tandem sequence repeats, which may represent a location of telomere healing. The proximity of short mTERC sequences to telomere healing was previously shown [23]. Thirteen sequences of the list matched the TERC boxH/ACA domain (position 350.6±2.5 to 385.5±10.8), which is known to be shared by other cajal body- associated non-coding RNA [24, 25]. To our surprise, one long sequence of 365nt, which we designated as alternative TERC (alTERC), showed an 87.9% similarity to mTERC as identified by the BLAT search. Comparing this sequence by global pairwise alignment to the known mTERC (397nt) revealed an 80.7% similarity. Importantly, a deletion of 18 bp in the CR4 region in the alTERC was observed (Figure 1B). To further examine whether the alTERC is a paralog of mTERC, we compared the alTERC to the Multiple Sequence Alignment (MSA) of TERC from 33 different mammals (for list of species see table in Supplement 1A and a FASTA file of the alignment in Supplement 1B). The results depicted in Figure 1C show that the alTERC is significantly (*P* < 0.001) more similar to the MSA consensus sequence of TERC than randomly generated sequences of the same length and nucleotide composition. It is important to note that alTERC is slightly less similar to the TERC MSA consensus sequence then the previously identified mouse or human TERC sequences, (Figure 1C).

### The alternative mouse TERC is expressed *in vitro* in mouse cell lines and *in vivo* in mouse brain and spleen

**Figure 2:**
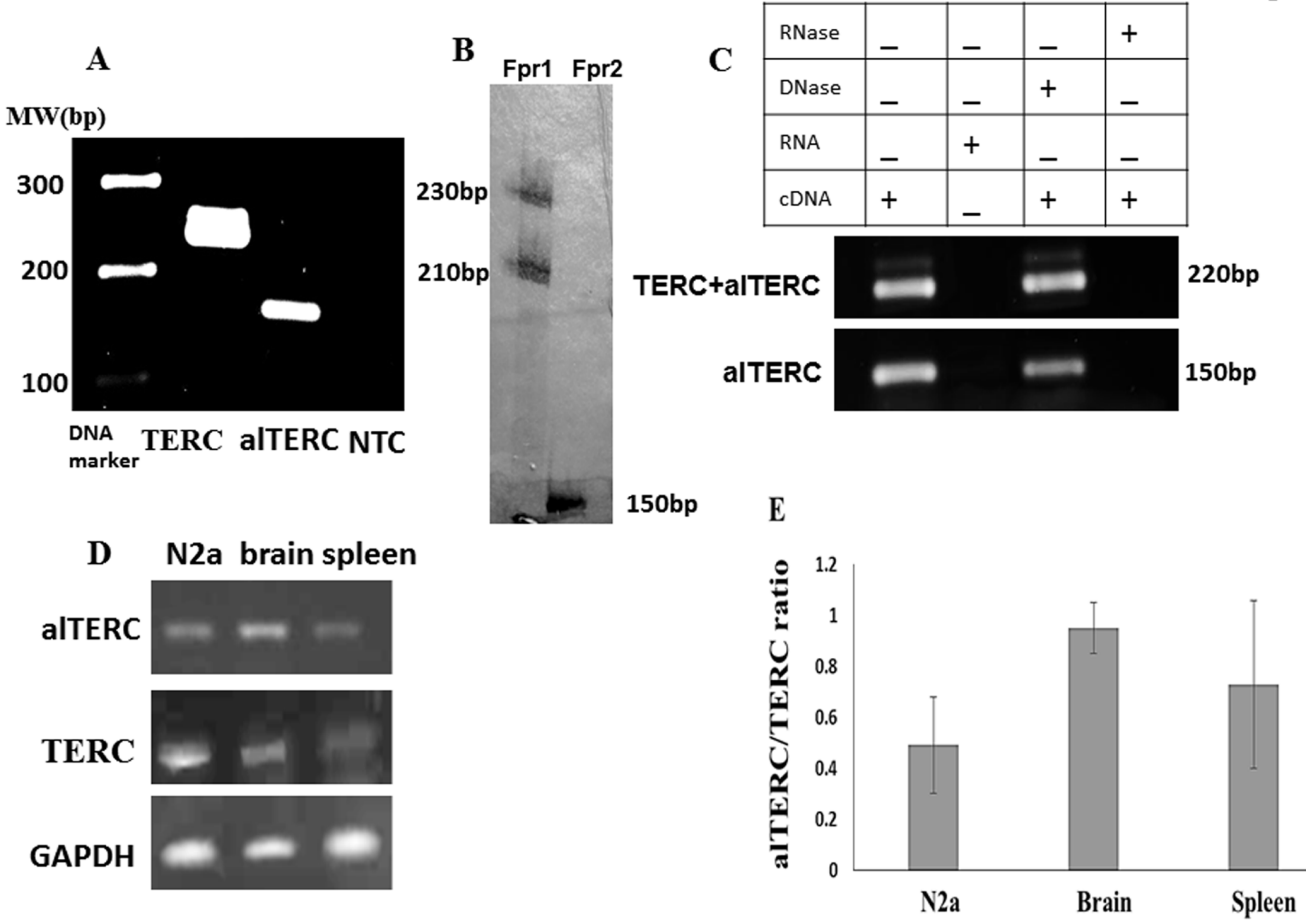
alTERC gene is transcribed to RNA *in vivo* in mouse organs and *in vitro* in mouse cell lines **A.** RNA was extracted from adult mouse brain, *n* = 3, cDNA was generated and subjected to PCR analysis using the set 1 primers for mTERC and set 2 primers for alTERC. Two bands of ~220 bp for mTERC and ~150bp for alTERC were observed. NTC- control, no cDNA. (A is a representative picture of 3 independent experiments). **B.** The PCR reaction described in A was carried out in the presence of radioactive nucleotide (dCTP [αp^32^] and the reaction products were analysed by 14% polyacrylamide gel electrophoresis following autoradiography. Two bands of ~230 bp and ~210 bp were observed with set 1 primers (Fpr1) for mTERC and one band of ~150 bp with set primers 2 (Fpr2) for alTERC were detected. **C.** RNA was extracted from mouse NSC-34 motor neurons like cells followed by cDNA production in the presence or absence of DNase or RNase and subjected to PCR amplification as described in A using the set1 and set 2 primers. **D.** RNA was extracted from mouse organs (brain and spleen) or from mouse neuroblastoma cell line (N2a) and subjected to sqPCR analysis using the set 1 and 2 primers for TERC and alTERC and GAPDH primers as control. A Representative picture of 3 independent experiments. **E.** The results of experiments described in D were quantified by densitometric analysis with the EZQuant software, calculated relatively to GAPDH and the alTERC/TERC expression ratio was estimated. The data are means ± SD of 3 independent experiments.

To examine whether the alTERC gene is transcribed to RNA, two sets of primers were designed: one that amplifies both TERC and alTERC and the second that amplifies only alTERC (see Figure 1B for primer positions and “Materials and Methods” for primers sequences). The high sequence similarity prevented the design of primers that are specific only to the mTERC. The first set of primers is predicted to amplify two products, 226bp from mTERC and 213 from alTERC and the second set of primers is predicted to amplify one product of the alTERC in a length of 148bp. The expression of alTERC in-vivo was examined in purified RNA samples of adult mouse brain that were treated with DNase (to rule out DNA contamination), followed by the generation of cDNA and the PCR amplification with the two specific primers. An amplification using TERC primers without template was carried out as control (NTC). The PCR products were separated on 1.5% agarose gel. The results show that two bands (not separated) of TERC (~220 bp) were produced by the first set of primers and one band of alTERC (~150bp) was produced by the second set of primers, indicating that the alTERC is indeed transcribed *in vivo* (Figure 2A). To separate between the products of primers set 1 and primers set 2, radioactive PCR was performed and the PCR products were analysed on 14% polyacrylamide gel electrophoresis. Figure 2B shows that indeed as predicted, the first set of primers amplified two products of about 230 and 210 bp and the second set amplified one 150 bp product. To confirm the specificity of the products and the primers, the PCR products were removed from the agarose gel (Figure 2A) and their sequence was determined. The sequence generated by primers set 2 matched perfectly with the alTERC genomic sequence (Supplement 2). However, since two bands of mTERC were generated by the first set of primers that were not separated on agarose gel, sequencing of these bands together resulted in a poor sequencing quality that partially matched to either TERC or alTERC. This is probably due to the mixture of the TERC and alTERC sequences produced by this set of primers.

To examine the expression of alTERC *in vitro*, in mouse neuronal cell culture (NSC-34), RNA was extracted from these cells, cDNA was prepared and PCR with the two sets of primers was performed. As expected the first set of primers produced the 220 bp band of TERC and the second set of primers the 150 bp band of alTERC. PCR products were not detected when RNA was added as the template or when pre-treatment with RNase (prior to cDNA production) was performed, and DNase treatment had no effect (Figure 2C). These results clearly show that in the mouse NCS-34 motor neuron like cell line, alTERC gene is transcribed to RNA. The presence of alTERC transcript was also demonstrated in the mouse neuroblastoma cell line (N2a) and in mouse spleen (Figure 2D). The ratio of alTERC to TERC was estimated as described in “Materials and Methods” section, and shows that the alTERC levels are lower than those of mTERC in all the examined organs and cell lines (Figure 2E).

### alTERC interacts with mTERT and hTERT

**Figure 3:**
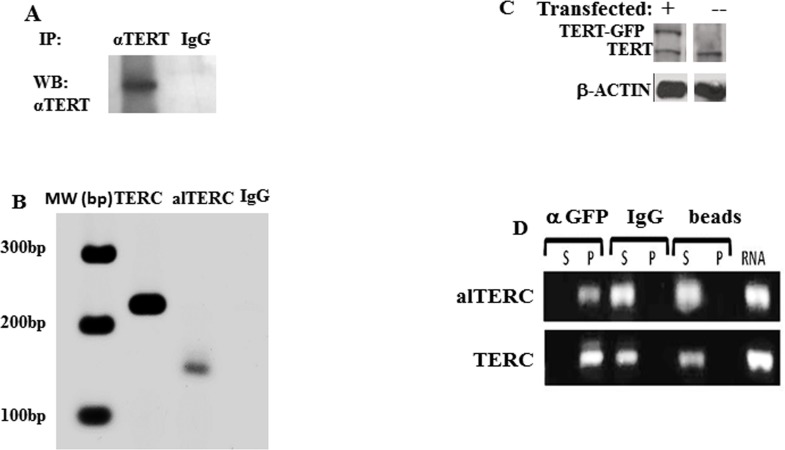
alTERC interacts with mTERT and hTERT **A.** Mouse TERT protein, derived from NSC-34 protein extract, was immunoprecipitated with anti-TERT antibody or unspecific IgG antibody. The immunocomplexes were subjected to Western blot analysis using anti-TERT antibody or **B.** subjected to RNA purification procedure followed by cDNA preparation and PCR with the mTERC or alTERC specific primers. **C.** Proteins extract derived from NSC-34^GFP-hTERT^ transduced cells and from NSC-34 cells were analyzed by Western blot with anti-hTERT antibody, in the transduced cells in addition to the endogenous mTERT the GFP-hTERT is also detected. **D.**Proteins extracts derived from the NSC-34^GFP-hTERT^ transduced cells was subjected to immunoprecipitation assay with anti- GFP or anti-IgG antibodies or precipitated with the protein A sepharose beads only as controlled. RNA was purified from the immune complexes (P) or from the supernatant (S), or from the transduced cells (RNA), cDNA was prepared and analysed by PCR using the alTERC and mTERC specific primers. Fig 3 is a representative picture of 3 independent experiments.

The potential interaction between the alTERC RNA and the mTERT protein was examined using similar methods described by Cifuentes-Rojas for the detection of the Arabidopsis TER2 [22, 26]. Immunoprecipitation of mTERT from the NSC-34 protein extract was performed using specific anti- TERT antibody [7, 27] and as a control the none-specific IgG antibody, instead of anti-TERT antibody, was used (Figure 3A). RNA was purified from the precipitated immunocomplexes and RT- PCR with the specific mTERC and alTERC primers was performed. The results depicted in Figure 3B revealed that mTERC and alTERC can be extracted from the mTERT/anti-TERT immunocomplexes but not from the none-specific IgG complexes, suggesting that the alTERC may interact with the endogenous mTERT protein.

It was previously shown that the hTERT protein can interact with the pseudoknot fragment of mTERC [28]. To determine if the mTERC and alTERC also interact with hTERT, cells (NSC-34) were transfected with a vector containing GFP-hTERT as previously described by us [7]. The expression of GFP-hTERT in these cells was also demonstrated (Figure 3C and [7]). The hTERT was immunoprecipitated with anti-GFP antibody and RNA was extracted from the immunocomplexes and from the supernatant as aforementioned. The presence of TERC and alTERC in the extracted RNA were detected by PCR amplification of the cDNA with the specific primers. The results depicted in Figure 3D show that the mouse TERC and alTERC were detected only in the anti GFP-hTERT immunocomplexes and were not detected in the pellet of the control samples (unspecific antibody or the protein A- sepharose beads). The mTERC and alTERC were, as expected, detected in the supernatant of the control samples. The results suggest that mouse TERC and alTERC can interact with the hTERT expressed in the NSC-34 cells.

### alTERC modified telomerase enzymatic reaction

**Figure 4:**
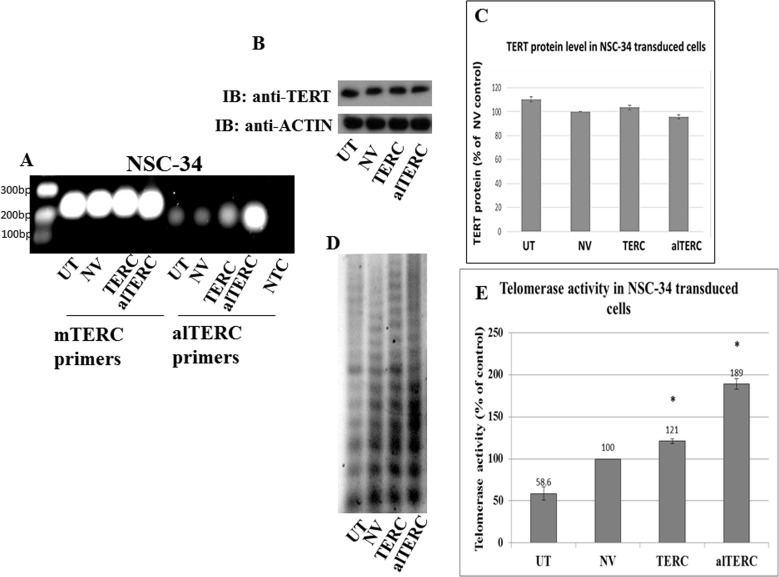
Stable overexpression of mTERC and alTERC increased telomerase activity in NSC-34 cells **A.** mTERC and alTERC were cloned into a retroviral vector and stable transduction of NSC-34 cells was performed. The expression of mTERC and alTERC in the transduced cells and in the control untransduced (UTr) or transduced with the empty vector (NV) cells were detected by PCR using the appropriate mTERC and alTERC primers. NTC- control without cDNA. **B.** TERT protein was detected by Western blot analysis with anti-TERT antibody and **C.** quantification of TERT protein relatively to the control β-actin protein was performed by densitometric analysis using the EZQuant software. The data are means ± SD of 3 independent experiments. **D.** Telomerase activity was measured by TRAP assay and, **E.** quantified by densitometric analysis using the EZQuant software. The results are % of the control NV and are means±SD, t Test, *p* < 0.05.

The interaction of alTERC with TERT suggests that it may regulate the telomerase enzymatic reaction. Therefore, alTERC and mTERC were cloned into a retroviral vector and stable transduction of NSC-34 mouse motor neuron like cells with both constructs was performed as described in “Materials and Methods”. To confirm the overexpression of these genes in the cells, PCR reaction with the two sets of primer was performed. The results depicted in Figure 4A demonstrate the overexpression of mTERC or alTERC in the transduced cells. Telomerase protein level was examined in the transduced cells by Western blot analysis and the results shown in Figure 4B and 4C revealed that no change in the TERT protein levels was detected. Telomerase activity was measured by the classical TRAP assay, with the modified detection recently reported by our group [29]. Figure 4B shows a representative image of telomerase activity in NCS-34 cells transduced with mTERC, alTERC or with the empty retrovirus vector (NV). Overexpression of mTERC or alTERC significantly increased telomerase activity compared to untransduced cells or cells transduced with the empty vector (Figure 4D). TERT activity in the alTERC overexpressing cells increased by 1.89 fold and in the mTERC overexpressing cells by 1.21 fold compared to the control cells (Figure 4E).This suggests that alTERC can support telomerase activity.

### TERC and alTERC overexpression protected NSC-34 cells from oxidative stress

**Figure 5:**
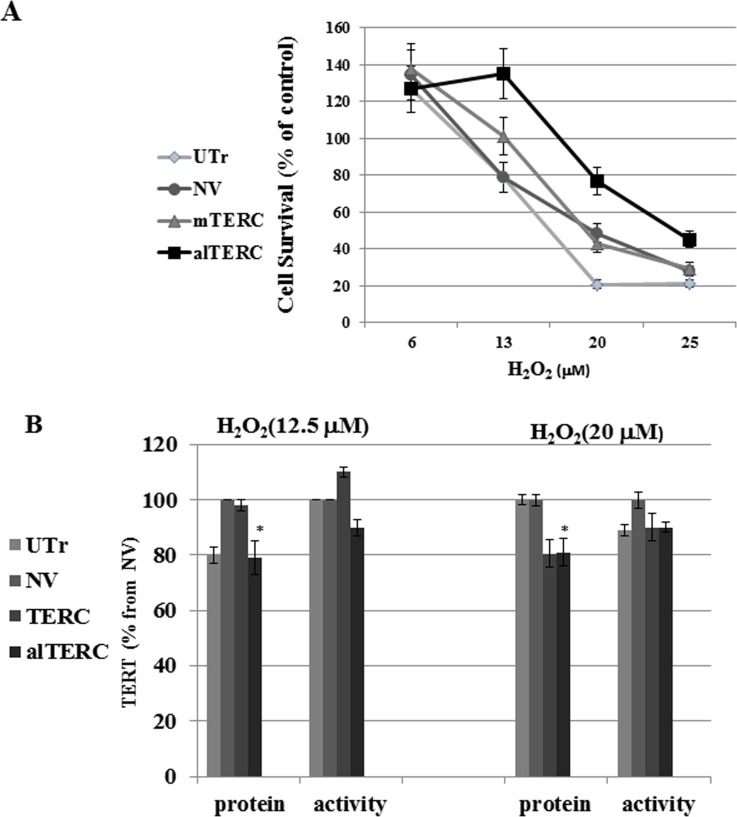
alTERC protects NSC-34 cells from oxidative stress without affecting TERT expression **A.** The alTERC or mTERC overexpressing NSC-34 cells were exposed to increasing concentrations of H_2_O_2_ in a FCS depleted medium for 4 hrs and then replaced by fresh FCS containing medium for 24 hrs following by cell cytotoxicity measurement using XTT assay. Cell survival as % from control H_2_O_2_ -untreated cells was determined. The results are means ±SD, symbols: UTr, untransduced NSC-34 cells; NV, NSC-34 cells transduced with the empty vector; alTERC, NSC-34 cells overexpressed alTERC; TERC, NSC-34 cells overexpressed mTERC. **B.** Protein extracts were prepared from the cells treated as described in A and subjected to Western Blot analysis with anti TERT antibody and anti β- actin antibody (as control) or to telomerase activity using TRAP assay. Quantification of TERT level or telomerase activity was performed as described in “Materials and Methods” section and the data are presented as % of the control NV-transduced cells. The results are means ±SD of 3 independent experiments, * *p* < 0.05.

To examine the effect of mTERC and alTERC overexpression on the survival of the cells following exposure to oxidative stress, the various transduced NSC-34 cells were exposed to different concentrations of H_2_O_2_ for 4 hrs., and cell survival was determined by the XTT assay after 24 hrs. The results depicted in Figure 5A demonstrate that overexpression of alTERC and mTERC in NSC-34 cells increased the survival of cells treated with H_2_O_2_ up to 4 fold (for alTERC) and 2 fold for mTERC (at 20μM H_2_O_2_) compared to H_2_O_2_ -treated but untransduced cells (UTr). The results suggest that TERC protects cells from oxidative stress and the alTERC was more potent than mTERC in this matter.

It was previously shown that TERT, in additional to its canonical activity (re-elongation of telomeres) possessed non-canonical properties such as protecting the mitochondria from oxidative stress [30]. Therefore, it is possible that under oxidative stress conditions the protective effect of mTERC or alTERC is due to their effect on the endogenous mTERT. We therefore examined the effect of overexpression of mTERC and alTERC on mTERT activity and under oxidative stress in NSC-34 cells.

The various transduced cells were exposed to different concentrations of H_2_O_2_ and TERT protein level and activity was examined. A slight increase/decrease of 10% in TERT activity and a 20% decrease in TERT protein level was observed in the mTERC or alTERC transduced cells exposed to 12.5 or 20 μM H_2_O_2_ (Figure 5B) suggesting that the protective anti oxidative effects of mTERC or alTERC is not due to the increase in TERT expression and activity in the transduced cells.

## DISCUSSION

TERC is one of the lncRNA that was shown to possess a biological function. Studies suggested that emergence of new lncRNAs may be the consequence of genome duplication and transposition [31]. Here we identified, first by bioinformatic tools and secondly by experimental procedures that the mouse genome contains an additional TERC gene which as the canonical mTERC, is located in chromosome 3. This additional TERC gene, designated alTERC, shows an 87.9% similarity to the canonical mTERT and possesses an 18 bp deletion in the CR4 region. Despite the high similarity between the canonical mTERC and the alTERC, we were able to design sets of primers that can distinguish between the mTERC and alTERC in the PCR assay. We showed that the alTERC is transcribed *in vivo*, in mouse brain and spleen and *in vitro* in mouse motor neurons and mouse neuroblastoma cell lines. However, in the mouse brain, the ratio alTERC/mTERC was almost equal, while in the spleen and in neuroblastoma the expression of alTERC was lower by 2 fold compared to the canonical mTERC.

To the best of our knowledge the present study is the first report of additional functional TERC gene in mammals. Variants of TERC were reported previously and some of them are processing intermediates [32] and others are considered to be pseudogenes [8]. However, in Arabidopsis thaliana the presence of an additional TERC gene (TER2) was previously demonstrated [22, 26]. The alTERC in mouse shows a high similarity to the canonical mTERC in all the CR regions, except CR4, while in Arabidopsis, the TER2 significantly differed from the canonical TERC. In Arabidopsis, both genes share a 220 nt highly conserved domain which in TER2 is divide into two segments interrupted by intervening sequences of 529 nt. Processing of the TER2 by splicing and 3′ end cleavage results in the formation of a third telomerase RNA component TER2_s_, which contains a contiguous 220 nt stretch with 85% identity to the corresponding region in TER1 [22]. Therefore, the alTERC that we identified in the mouse genome is probably due to gene duplication of TERC while TER2 is a consequence of transposable element insertion and gene duplication [31]. The data also show that the endogenous mTERT binds and interacts with the alTERC. However only a small portion of TERT actually interacts with the alTERC since a relatively faint band of alTERC compared to TERC was detected (see Figure 3) despite the strong band of alTERC observed in the expression analysis (Figure 2), suggesting that the interaction of mTERT with the canonical mTERC is preferred over the interaction with the alTERC. alTERC, as well as the canonical mTERC, were also purified from the hTERT/anti hTERT GFP immunocomplexes, demonstrating the possible binding and interactions between human TERT and mTERC and the ability of the mouse alTERC to interact with human TERT. Previous reports demonstrated that mTERC was unable to reconstitute telomerase activity with hTERT protein in an *in vitro* system but the interaction of hTERT with the pseudoknot fragment of mouse TERC was demonstrated [24]. Nevertheles, we show that in the cells hTERT can interact with mTERC. The hTERT also interacts more efficiently with the canonical mTERT rather than with the alTERC (Figure 3B) which has a deletion of 18 bp in the CR4 region.

CR4/CR5 is the trans-activation domain which is catalytically essential TERC element, that like the core domain, interacts directly with TERT [33]. Therefore the missing nucleotides in the CR4 domain of the alTERC may influence its binding affinity to TERT but will not diminish it.

**Figure 6:**
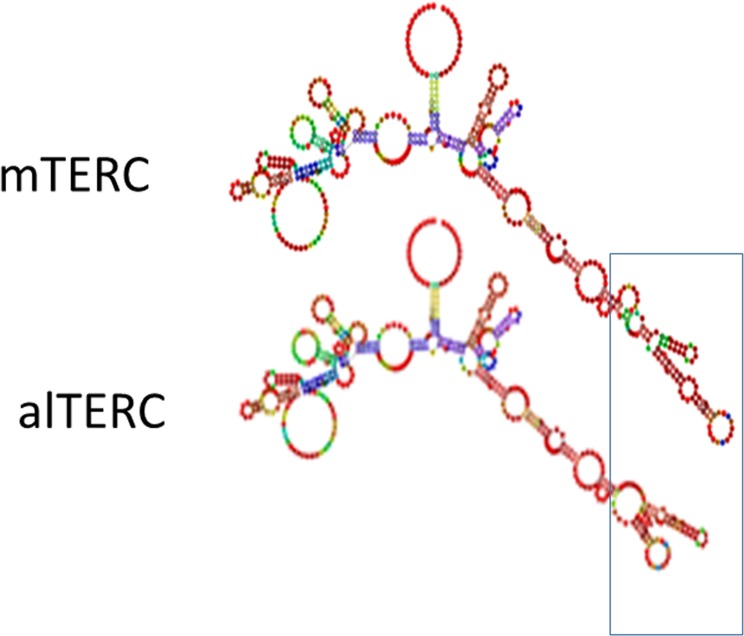
Predicted secondary structure of alTERC TERC MSA was manually modified to resemble to alTERC sequence (see supl 3) and the Alofold software was used for the secondary structure prediction of the alTERC in comparison to that of the predicted mTERC.

To predict the alTERC RNA secondary structure. TERC MSA was manually modified to resemble to alTERC sequence (Supplement 3 FASTA File) and the resultant MSA was used for RNA structure prediction using the Alofold software (Figure 6). The predicted structure of TERC is shown and resembles its published structure [24, 25] aside from the pseudoknot domain, which is frequently problematic for prediction by algorithms [34]. The main difference between the predicted alTERC and the predicted regular mTERC is a small stem loop in the co-activation domain highlighted in Figure 6. This predicted structure is the result of 18-nucleotide deletion in the alTERC conserved region 4. This small stem loop was shown to participate in TERT binding and in regulating telomerase enzymatic activity [13].

It was previously shown that the CR4/CR5 domain of TERC stimulates telomerase activity and binds to the Telomerase RNA binding domain (TRBD). The CR4/5 forms a three-way junction structure with flanking P5, P6, and P6.1 stem-loops and the p6.1 is required for TERT binding and telomerase activity [13, 25]. From the predicted secondary structure of the alTERC (Figure 6) it seems that the long stem loop structure of the CR4/CR5 was changed and one may expect that this may influence the binding ability of the alTERC to TERT compared to the canonical mTERC, as indeed was observed in the present study.

However, overexpression of mTERC or alTERC in NSC-34 cells increased telomerase activity by 1.2 fold for mTERC and 1.89 fold for alTERC but no significant effect on TERT protein level was observed, suggesting that this specific deletion in the CR4 region did not reduce the catalytic activity of TERT. TERC was previously shown to possess an anti-apoptotic role in human immune cells independent of its function in telomerase activity [21]. To test the biological function of the alTERC in the motor neurons cells we examined the effect of overexpression of alTERC and the canonical mTERC on cell survival following exposure to oxidative stress. alTERC and mTERC significantly increased the survival of the motor neurons like cells by 2 and 4 fold respectively. alTERC protected motor neurons cells from oxidative stress more efficiently then the canonical mTERC and this protecting effect was not due to the increase in TERT expression or activity. It was previously reported that overexpression of enzymatically inactive TERC can protect T cells from dexamethasone induced apoptosis [21]. Here we show that overexpression of alTERC with a small deletion in the CR 4 region conferred resistance to the cells from oxidative stress without increasing TERT expression or activity, although under normal conditions this alTERC is enzymatically active. We previously showed that it is possible to efficiently protect mouse motor neurons cells and human mesenchymal stem cells from oxidative stress by increasing TERT expression and activity with telomerase activating compounds AGS-499 and AGS-500 which specifically increased TERT expression [7, 27] but do not influence TERC expression (unpublished data). This suggest that in addition to the non-canonical function of TERT in protecting cells from oxidative stress, the presence of the alTERC gene in the mouse genome and its expression *in vivo* may provide an additional pathway for regulating telomerase activity under natural state and protecting cells and tissue under oxidative stress conditions.

## MATERIALS AND METHODS

### Cells

mouse NSC-34, a motor neuron like cell line [35], was kindly received from Dr. Daniel Offen, Tel-Aviv University, Israel. Neuro-2a: mice neuroblastoma cell line was purchased from ATCC (CCL-131). GP2-293 packaging cells: (Clontech Laboratories, Mountain View, CA, USA) are Human embryonic kidney (HEK) cells transformed with adenovirus type 5 DNA and stably expressed the viral *gag* and *pol* protein.

All cell lines were cultured in DMEM supplemented with 10% FCS, 1% L-glutamine, 1% penicillin-streptomycin and for the NCS-34 1% non-essential amino acid was also added.

### Animals

male mice CD-1 (1-6 months) were purchased from HARLAN Laboratories INC. (Jerusalem, Israel). All animal procedures were approved by the animal experimentation ethics committee at Ben-Gurion University (IL-39-08-10, IL-07-06-14).

### Antibodies

Rabbit monoclonal anti-TERT antibody (ab32020, abcam, Cambridge MA, USA) were used for immunoprecipitation of mTERT, and anti TERT monoclonal antibody (1531-1, Epitomic, CA) for Western blot analysis. anti-GFP antibody (B-2, SC-9996 Santa Cruz Biotechnology, USA) was used for immunoprecipitation of hTERT-GFP. Mouse monoclonal Anti- β-actin antibody (clone C4, MP Bio-medicals, USA) for detection of β-actin by Western blot procedure

### Plasmids and expressing vectors

pGEM T-Easy vector (Promega, USA), BABE-hygroHighe-GFPhTERT vector containing hTERT-GFP (Addgene, Cambridge, MA USA), Custom made retroviral plasmid NV-5119 was based on pMSCVneo (Clontech). The PGK promoter driving expression of the Neo resistance gene has been removed and IRES inserted instead; the multiclonal site has been expanded to include more restriction sites.

### Purification of total RNA from mouse brain and from NSC-34 cells

Mouse organ (brain or spleen) was quickly removed and washed with Ringer solution followed by homogenisation in Tri-reagent RNA extraction buffer (Sigma-Aldrich, Rehovot, Israel) and RNA was isolated according to the manufacture's protocol. Cells were washed twice with sterile PBS and scraped in PBS buffer prior to centrifugation at 700 x g for 10 minutes. The supernatant was removed and the cell pellet was re-suspended in Tri-reagent. In both cases DNA residues were removed using the “DNase I, RNase-free” kit (Thermo Fisher Scientific Inc, Pittsburgh PA, USA). RNA concentration was determined using NanoDrop 2000c spectrophotometer (Thermo Fisher Scientific Inc., Pittsburgh PA, USA). The RNA was transcribed to cDNA with the “Revert Aid First Strand cDNA Synthesis Kit” (Thermo Fisher Scientific Inc, USA) according to the manufacturer's instructions.

### TERC and alTERC identification by PCR

Specific PCR primers to detect alTERC and mTERC were designed:

Set 1: mTERC Fw- GTTTTTCTCGCTGACTTCCAG, mTERC Rv- CGCCCCGCGGCTGACAGAG;

set 2: alTERC Fw- GGTCCCGTGCCTCAAGAAAC, alTERC Rv-CGCCCCGCGGCTGACAGAG.

TERC primers (set1) amplify the mTERC which is the mouse known TERC (226bp) and the alTERC (213bp), alTERC primers (set 2) are predicted to amplify only the alternative TERC and should generate an amplicon in the size of 148bp. The products were separated on 1.5% agarose gel and stained using ethidium bromide. The products were extracted from the gel using Gel Extraction kit (Qiagen, Germany) and sequenced to confirm the correct gene fragment amplification.

### Preparation of mTERC and alTERC expressing vectors and stable transduced NSC-34 cells

mTERC and alTERC genes were amplified by PCR using the following primers : TERC Fw: AGGCCTCGGCACCTAACCCTGA; TERC Rv: CAGCGGGAATGGGGGTTGTG;

alTERC Fw: TGTGGCCTGTGTCTAACCCTGC; alTERC Rv: GGTGCACTTCCCGCAGCTCA.

The mTERC 420 bp and alTERC 377 bp products were separately inserted into a pGM plasmid and transfected to E.coli grown in the presence of ampicillin [0.1mg/ml]. The plasmids were extracted from E.coli using Gene JET Plasmid Miniprep kit (Thermo scientific, USA) following by PCR amplification, agarose gel analysis, extraction of the DNA products from the gel and verification by sequencing. Retroviral expressing vectors containing the TERC and alTERC genes were prepared by cloning of these genes into custom made retroviral plasmid NV-5119 (described above) and transfected into XL-10 Gold ultra-competent bacteria (Agilent/Stratagene). The plasmids were purified from the different bacterial colonies using the GeneJET Plasmid Miniprep Kit (Thermo) and the extracted DNA was analysed for the presence of TERC and alTERC by using restriction enzyme, EcoRI, according to the digesting map, and the correct constructs were verified by DNA sequencing. The constructs were sub-cloned into bacteria DH-5α to increase the yield of the plasmid.

### Preparation of NSC-34 cells stably transduced with mTERC or alTERC genes

To produce infecting retroviral expressing vectors, GP2-293 packaging cells (Clontech) were co-transfected with the above described TERC or alTERC constructs and with pVSV-G (Clontech). The medium containing the retroviral particles was used for the infection of NSC-34 cells. Cells containing the TERC or alTERC expressing vectors were selected by growing the infected NSC-34 cells with G418.

### Preparation of whole cell proteins extracts from mouse brain and NSC-34 cells

Whole cell protein extracts from mouse brain and from NSC-34 cells were prepared as previously described by us [7, 29]. Briefly: The brain was homogenized in ice using a manual homogenizer (pestle B). NSC-34 cells were harvested, pelleted by centrifugation, washed by PBS solution and re-suspended in PBS. Mouse brain homogenates and NSC-34 cell suspension were pelleted by centrifugation (500 g at 4°C). The cell and tissue pellets were re-suspended in CHAPS lysis buffer (10mM Tris-HCl pH-7.0, 1mM MgCl_2_, 1mM EDTA pH-0.5, 0.1mM PMSF, 0.5% 3 [(3 Cholamidopropyl) dimethylammonio]-propanesulfonic acid [CHAPS] and 10% glycerol, and kept on ice for 30 min. Mechanical lysis was performed by passing the solution 10-15 times through a 1ml syringe with a 21G needle. The protein fraction was obtained by centrifugation at 13000g at 4°C for 30 min and the supernatant was collected.

### TRAP assay for measuring telomerase activity

Evaluation of telomerase activity was performed using the modified TRAP assay developed in our lab [29]. Protein extracts (1μg/μl) were incubated with 2μl of 10X TRAP assay reaction mix (20mM Tris-HCl pH 8.2, 63mM KCl, 1.5mM MgCl2, 1mM EDTA, 0.1mg/mL BSA and 0.05% Tween 20 Dissolved in Ultra-Pure H_2_O_2_ [UPW]), 1μl of telomerase substrate (TS) primer (5′-AATCCGTCG AGC AGA GTT-3′, 0.1μg/μl), 1μl of 10mM dNTP's mix (Sigma) and 15μl UPW for 45 minutes at 30°C in a water bath. The telomerase reaction products were mixed with PCR reaction mix containing 1μl ACX reverse primer (5′-GCG CGG CTT ACC CTT ACC CTT ACC CTA ACC-3′, 0.1μg/μl), 2μl UPW, 2.6μl Titanium Taq-polymerase buffer and 0.4μl Titanium Taq-polymerase (Clontech Laboratories, Inc. USA) and inserted to a PCR thermo-cycler at the following program: 90°C - 2 minutes, 34 cycles of (94°C - 30 seconds, 50°C - 30 seconds, 72°C - 45 seconds), 72°C - 2 minutes. The PCR products were separated on a 4.5% mini-gel high-resolution agarose (Sigma-Aldrich Ltd, Israel) at 4°C (cold-room) and at 110v for 3 hours and the telomerase specific DNA products were stained with the highly sensitive nucleic acid stain Gel-Red (Biotium Inc. USA, X10000 stock solution diluted to X3 working solution with UPW) for 20 minutes. The gels were filmed using a UV trans-illuminator digital camera system at 302nm wavelength.

TRAP assay products were quantified by densitometric analysis using the EZQuant software for analysis of 1D gels (EZQuant Ltd. Tel-Aviv, Israel) [7, 27]

### TERT immunoprecipitation, and isolation of TERC from the immunocomplexes

Protein extracts derived from NSC-34 cells (300 μg) were incubated (over-night) with monoclonal anti-TERT antibody, protease inhibitors mixture and CHAPS buffer at 4°C in revolving rotator. Protein A/G agarose beads in CHAPS buffer were added and incubated for 2h in 4°C in revolving rotator. Tubes were centrifuged at 17,000g for 5min, supernatant was removed and beads were washed three times with 400μl CHAPS buffer. The samples were than analysed by Western blot for TERT precipitation and for the presence of TERC or alTERC by RNA purification from the immunocomplexs using the Tri-reagent RNA extraction Kit (Sigma-Aldrich, Rehovot, Israel).

#### Detection of TERT protein by western blot analysis

Whole cell proteins extracts (25 μg) were subjected to Western blot analysis as previously described. The antibodies used in this analysis: anti hTERT antibody (1:1250), anti-βactin antibody (1:10000) as primary antibodies. HRP conjugated anti- rabbit (1:5000, NA934, GE Healthcare, USA) and anti-mouse (1:10000, SC-2005, Santa Cruz Biotechnology, USA) as secondary antibodies respectively. The immunocomplexes were detected using the enhanced chemiluminescence (ECL) kit (Biological Industries, Beith Haemek, Israel) and medical X-ray film (Fujifilm).

## SUPPLEMENTARY MATERIAL


