# Supplementary material for: Expression of functional alternative telomerase RNA component gene in mouse brain and in motor neurons cells protects from oxidative stress
Source: Oncotarget. 2016 Nov 3;7(48):78297–309. doi: 10.18632/oncotarget.13049 (PMC5346639; doi:10.18632/oncotarget.13049)
Supplement: Supplementary file 1 [file oncotarget-07-78297-s001.pdf]

Expression of functional alternative telomerase RNA component gene in mouse brain and in motor neurons cells protects from oxidative stress

Supplementary Material

Supl 1A: the alTERC was compared to Multiple Sequence Alignment (MSA) of TERC from 33 different mammals:

Mammals name	
Oryctolagus cuniculus(rabit)	Microtus ochrogaster (hedgehog)
Callithrix jacchus (monky)	Cricetulus griseus (hamster)
Macaca mulatta (advence monky)	Mus musculus (mouse)
Nomascus_leucogenys (advence monky)	Mus spretus (mouse)
Pongo_pygmaeus_abelii (primate)	Mus musculuscastaneus (mouse)
Gorilla_gorilla_gorilla (primate)	Rattus norvegicus (rat)
Homo sapiens (primate)	Mustela putorius furo (samor)
Pan_troglodytes_TERC (primate)	Procyon lotor (racoon)
Ovis_aries_TERC (sheep)	Felis catus (cut)
Bos taurus (cow)	Muntiacus muntjak vaginalis (gaseal)
Equus caballus(horse)	Sus scrofa (pig)
Canis_lupus_familiaris (dog)	Suncus murinus (mouse)
Ailuropoda_melanoleuca (panda)	Dasypus novemcinctus (armadilo)
Tupaia glis belangeri (squirrel)	Dasyurus hallucatus (cat)
Cavia porcellus (guiina pig)	Elephas maximus (elphent)
Chinchilla brevicaudata (chancialw)	Trichechus manatus (see cow)
Geomys breviceps (digger)	

Supl. 1B: the result of the comparison of alTERC to the MSA of TERC from the various mammals is provides as a FASTA file.

Supl. 2: The sequence generated by primers set 1 showed 65.5% identity to the genomic TERC sequence

```
   65.5% identity in 206 nt overlap (55-259:1-201); score:  307 E(10000)

               60        70        80        90       100       110
terc    TGTTTTTCTCGCTGACTTCCAGC-GGGCCAGGAAAGTCCAGACCTGCAGCGGGCCACCGC
        ::::::::::::::  ::    : ::::::  :::::  :  :    ::   :   : ::
TERC    TGTTTTTCTCGCTGTGTTTTTACTGGGCCATTAAAGTGTACCCGGAAAGTCCGGACCTGC
                10        20        30        40        50        60

              120       130       140       150       160       170
terc    GCGTTCCCGAGCCTCAAAAACAAACGTCAGCGCAGGAGCTCCAGGTTCGCCGGGAGCTCC
          ::: ::  ::::::: :::: ::     : :: ::   : : ::::::   :: :: :
TERC    AAGTTGCCACGCCTCAATAACATACTGTGCCTCAAGAAACCTAAGTTCGCGCAGA-CTTC
                70        80        90       100       110

              180       190       200       210       220       230
terc    GCGGCGCCGGGCCGCCCAGTCCCGTACCCGCCTACAGGCCGCGGCCGGCCTGGGGTCTTA
        :::: ::   :::  :::::  ::  :: ::  ::::::::  ::: ::::::: ::: :
TERC    GCGGTGCGCCGCCAGCCAGTGGCGCCCCGGCGCACAGGCCGGTGCCCGCCTGGG-TCT-A
      120       130       140       150       160       170

              240       250
terc    GGACTCCGCTGCCGCCGCGAAGAGCT
        ::: ::::::::: : :  :::::::
TERC    GGA-TCCGCTGCCAC-GGTAAGAGCT
        180        190        200
```

The sequence generated by primers set 2 showed 95.2 % identity to the alTERC (paralog) genomic sequence

```
   95.2% identity in 126 nt overlap (145-270:2-124); score:  534 E(10000)

             150       160       170       180       190       200
paralo GGTCCCGTGCCTCAAGAAACGTCAGCGCGCAGGCGCTCCAGGTTTGCCCGGAGCCCGC
       ::::::::::::::::::::::::::::::::::::::::::::::::::::::::::::
Paralo GGTCCCGTGCCTCAAGAAACGTCAGCGCGCAGGCGCTCCAGGTTTGCCCGGAGCCCGC
               10        20        30        40        50        60

             210       220       230       240       250       260
paralo GGCGCCCGGGCGCCCCGCCCGGTGCCCGCCTGGGGTCTTAGGACTCCGCTGCCACCGGGA
       :::::::::::::::::::::::::::::::::: ::: ::::: ::::::::::::  :
Paralo GGCGCCCGGGCGCCCCGCCCGGTGCCCGCCTGGG-TCT-AGGAC-CCGCTGCCACCGAAA
               70        80        90         100        110

             270
paralo AGAGCT
       ::: ::
Paralo AGAACT
     120
```

Supl 3. TERC MSA was manually modified to resemble to alTERC sequence presented here as FASTA file
